# Joint improvements of radar/infrared stealth for exhaust system of unmanned aircraft based on sorting factor Pareto solution

**DOI:** 10.1038/s41598-021-87756-0

**Published:** 2021-04-15

**Authors:** Ze Yang Zhou, Jun Huang

**Affiliations:** School of Aeronautic Science and Engineering, Beihang University, Beijing, 100083 China

**Keywords:** Aerospace engineering, Electrical and electronic engineering, Optical physics

## Abstract

In order to reduce the radar cross section (RCS) of the unmanned aircraft while suppressing its infrared signature, a comprehensive design method (CDM) based on sorting factor Pareto solution is presented. The physical optics and physical diffraction theory are used to evaluate the electromagnetic scattering characteristics of the aircraft, and the Monte Carlo and ray tracing method are used to evaluate the infrared radiation intensity of the exhaust system. CDM is used to evaluate and screen each individual in each offspring, and the design parameters and sub-models of the aircraft exhaust system are continuously improved. The results show that the exhaust port model, lower baffle and nozzle height are the main factors affecting the RCS indicators, nozzle stages, exhaust port model, lower baffle and outer width make the main contribution to infrared radiation suppression. The presented CDM is efficient and effective in enhancing the radar/infrared integrated stealth performance of the aircraft.

## Introduction

The exhaust system of an unmanned fighter not only affects its aerodynamic characteristics and infrared (IR) signature, but also affects the electromagnetic scattering characteristics of its tail and sides^1–3^. Studying the comprehensive design of the exhaust system is of great significance to the infrared/radar stealth performance of the aircraft.

Future fighters and bombers are inclined to ultra-flat fuselage layout, which also brings certain challenges to the design of their intake and exhaust systems, while the balance and restriction between multiple performances is also the key concern of interdisciplinary^4,5^. Computational fluid dynamics (CFD), physical optics (PO), radial basis functions and sequential quadratic programming are integrated to optimize the aerodynamic/stealth characteristics of the flying wing aircraft, and good design variables and strategies are obtained^6^. Diverter less technology and tail nozzle baffles are often used on stealth fighters to improve the aerodynamic and stealth characteristics of these aircraft^7,8^. Compared with the traditional layout, the flying wing layout has the advantages of high aerodynamic efficiency, stealth and internal space utilization^9,10^, which makes the design of its exhaust system must take into account the low radar cross-section (RCS) and infrared radiation. Surface pixel method, PO and equivalent electromagnetic flow method are used to solve the RCS of three-wing fighter and stealth fighter^11,12^. Based on the existing aerodynamic design concepts and RCS analysis methods, the stealth development of the unmanned fighter exhaust system will pay more attention to infrared radiation suppression and radar/infrared integrated stealth.

The rapid development of high-sensitivity, high-resolution infrared imaging detection technology has made the survivability of fighters with radar stealth design also challenged^13,14^. Replace the axisymmetric nozzle with a binary non-axisymmetric nozzle with larger width and height to obtain better infrared radiation suppression. Combining the exponential broad band model and the radiation transfer equation (RTE), an infrared radiation characteristic estimation method was developed to evaluate the infrared signature of the engine exhaust system^15^. Using the CFD method, the temperature field of the unmanned combat aircraft in the non-forced state is obtained, and the numerical calculation of infrared radiation is performed^16,17^. Ray tracing method is used to solve the infrared signature of high temperature exhaust gas from different nozzles^18^. The serpentine nozzle can effectively suppress the infrared radiation characteristics while enhancing the radar stealth characteristics of military aircraft^19^. With the rise of comprehensive stealth research^20^, the design and optimization of unmanned combat aircraft are more inclined to consider multiple performance^21,22^. The gray correlation model can effectively evaluate the aerodynamic characteristics, RCS and IR signatures of the aircraft^22–24^. The comprehensive optimization method based on Pareto solution can also give a better solution to the needs of many aspects of stealth^25,26^. The continuous development of various technologies of unmanned bombers^27^ has also prompted its radar stealth design and infrared radiation suppression to achieve a better balance.

Previous research on the exhaust system of unmanned combat aircraft has mostly focused on aerodynamic shape improvement and infrared radiation characteristic calculation. With the continuous development and joint search of radar and infrared detectors, unilateral reduction of RCS or IR signatures has been unable to counter the threat of multiphysics. In view of the mutual constraints of aerodynamics, radar stealth and infrared radiation suppression when designing the unmanned aircraft exhaust system, this paper attempts to establish a hybrid design approach to establish a comprehensive stealth scheme that takes into account aerodynamic characteristics. This is of guiding significance and engineering value for improving the survivability and integrated combat effectiveness of unmanned fighters.

In this manuscript, the comprehensive design method (CDM) is presented in Sect. 2. Models of unmanned aircraft and exhaust system are built in Sect. 3. The results of RCS and IR signatures are provided and discussed in detail in Sect. 4. Finally, the full article is summarized in Sect. 5.

## Comprehensive design method

**Figure 1:**
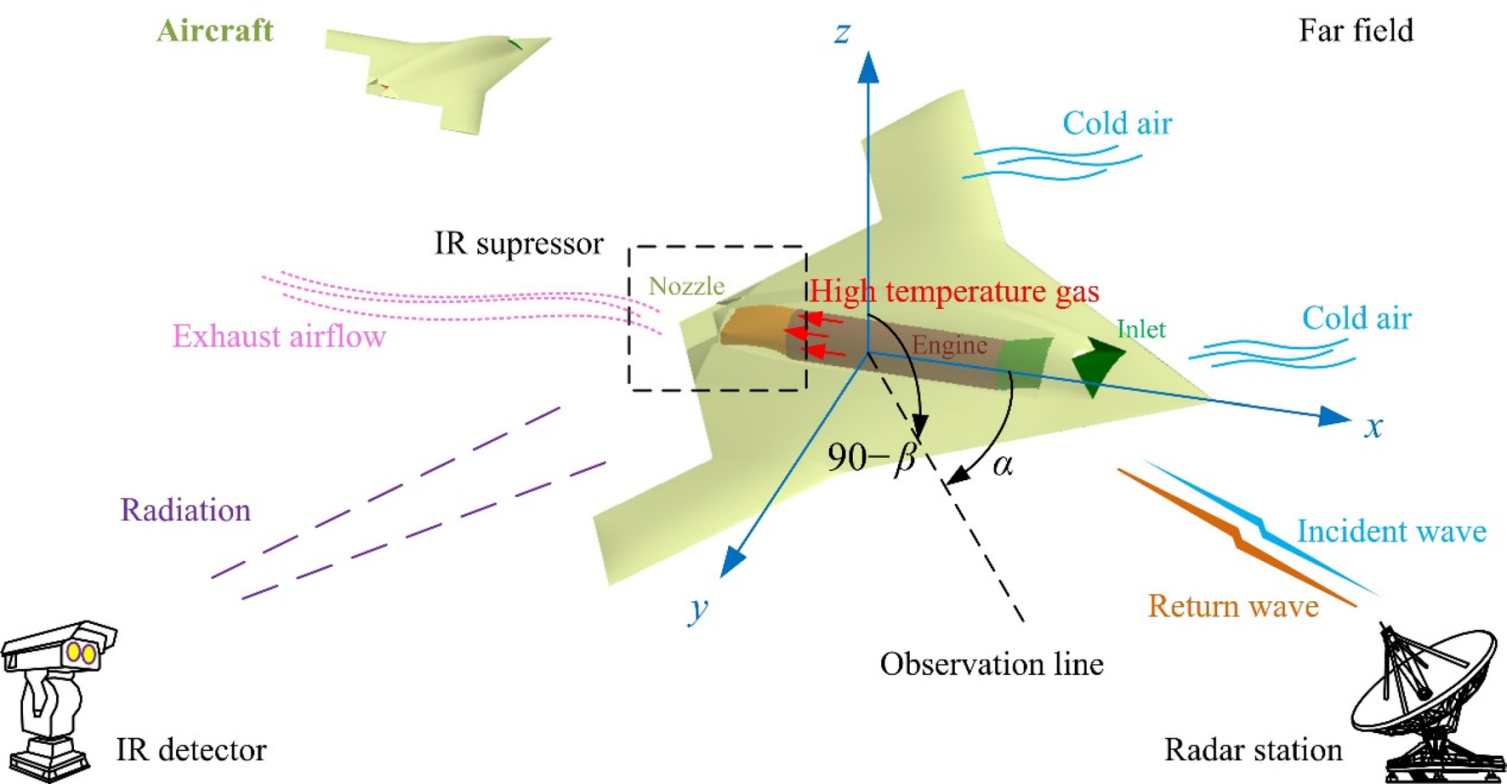
Schematic diagram of radar/infrared integrated stealth design of aircraft exhaust system, drawn using Microsoft Visio 2007.

The analysis of the threat of unmanned aircraft from radar and infrared detectors is shown in Fig. 1, where *α* is the azimuth between the radar station and the aircraft, *β* is the elevation angle between the radar station and the aircraft. The comprehensive design method is used to reduce the electromagnetic scattering level of the aircraft exhaust system while suppressing its infrared radiation.

**Figure 2:**
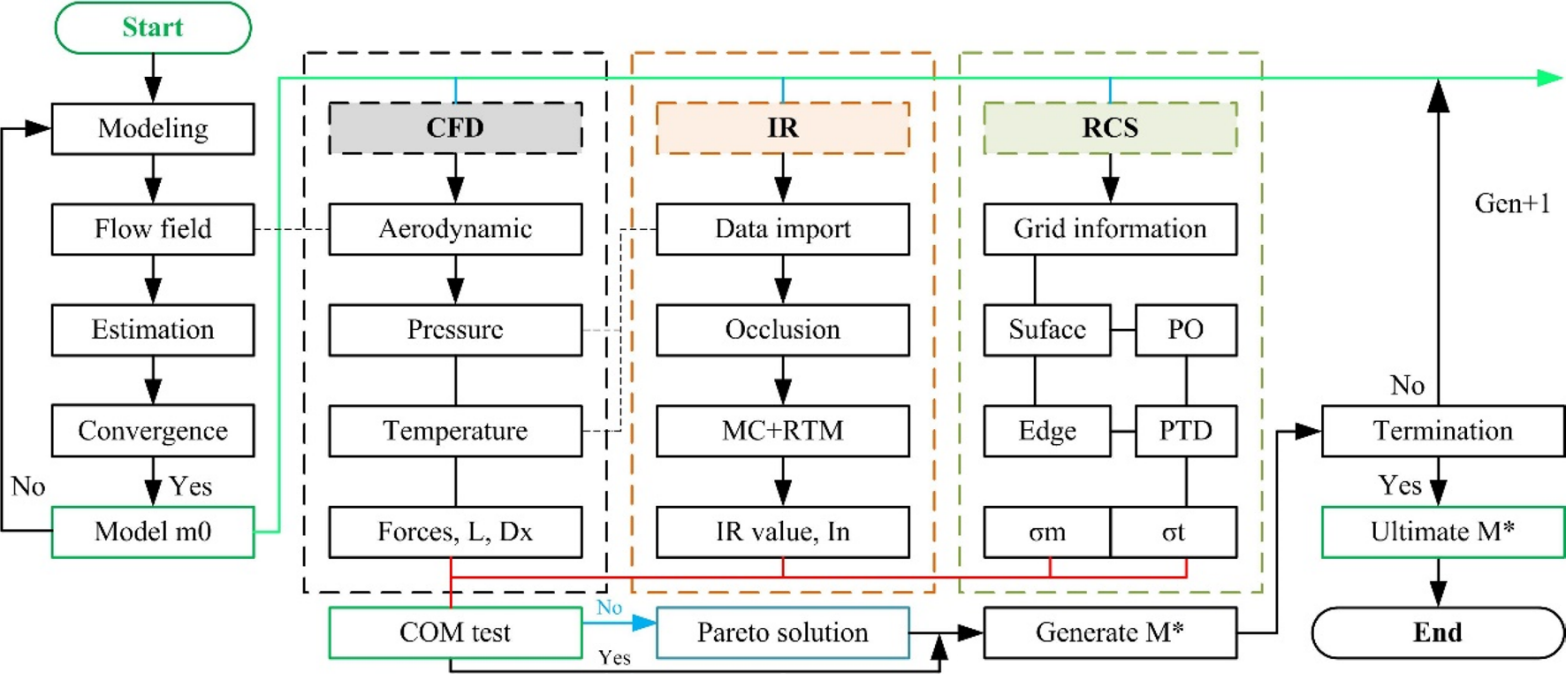
Flow chart of the comprehensive design method, drawn using Microsoft Visio 2007.

The entire design process includes three parts as shown in Fig. 2: the establishment of the initial model (*m*_0_), the calculation of performance indicators and the cycle judgment module. The first part emphasizes that both the flow field calculation and the grid need to converge to ensure that the subsequent calculations are comparable. The middle module performs aerodynamic, IR and RCS evaluation on each individual in each offspring, and comprehensively evaluates the optimal solution (M*). The third part judges the termination of the obtained M* to see if the next generation (Gen) design is needed.

### RCS calculation

PO and physical theory of diffraction (PTD) are used to solve the electromagnetic scattering characteristics of each individual in each offspring^3^. According to the magnetic vector position caused by the induced current on the surface of the target model, the electric field and magnetic field can be obtained as follows:

1 $$  {\text{E}}\left( {\text{r}} \right) = \frac{1}{{{\text{j}}\omega \varepsilon  \cdot 4\uppi }}\iint\limits_{{S^{\prime } }} {\left[ {\frac{{3 - k^{2} R^{2}  + j3kR}}{{R^{5} }}e^{{ - {\text{j}}kR}} {\text{R}} \times \left( {{\text{R}} \times {\text{J}}_{s} \left( {{\text{r}}^{\prime } } \right)} \right) + 2{\text{J}}_{s} \left( {{\text{r}}^{\prime } } \right)\frac{{1 + {\text{j}}kR}}{{R^{3} }}{\text{e}}^{{ - {\text{j}}kR}} } \right]}{\text{d}}S^{\prime }   $$

2 $$  {\text{H}}\left( {\text{r}} \right) = \frac{1}{{4\uppi }}\iint\limits_{{S^{\prime } }} {\frac{{ - 1 - jkR}}{{R^{3} }}e^{{ - {\text{j}}kR}} \left( {{\text{R}} \times {\text{J}}_{s} \left( {{\text{r}}^{\prime } } \right)} \right){\text{d}}S^{\prime } }  $$

where ***r*** is the coordinate vector of the field point, *ω* is the electromagnetic wave angular frequency, *k* is the wave number in free space, ***R*** is the distance vector between the field point and the source point, ***r***′ refers to the coordinate vector of the source point, *ε* is the dielectric permittivity^28–30^, ***J***_s_ represents the induced current on the target surface. When calculating RCS, the integral surface of the model is treated as an ideal conductor.

According to the assumption of PO, there is current in the illuminated area of the target surface, but no current in the dark area:

3 $$ {\text{J}}_{s} = \left\{ {\begin{array}{*{20}l} {2{\text{n}} \times {\text{H}}} \hfill & {Z_{\text{I}} } \hfill \\ {\mathbf{0}} \hfill & {Z_{D} } \hfill \\ \end{array} } \right. $$

where *Z*_I_ refers to the illuminated area, *Z*_D_ is the dark area, and ***n*** represents the unit normal vector of the outer normal direction of ***r'*** at the surface of the scatterer. Therefore, the electric field formula can be changed to

4 $${\text{E}}^{s} \left( {\text{r}} \right) = \frac{{\text{j}}}{{\lambda {\text{r}}}}\left| {{\text{E}}_{0} } \right|{\text{e}}^{{ - {\text{j}}k \cdot {\text{r}}}} \iint\limits_{{S^{\prime } }} {{\hat{\text{r}}} \times \left\{ {{\hat{\text{r}}} \times \left[ {\left( {{\hat{\text{n}}}\left( {{\text{r}}^{\prime } } \right) \cdot {\text{E}}_{0} } \right){\hat{\text{k}}} - \left( {{\hat{\text{n}}}\left( {{\text{r}}^{\prime } } \right) \cdot {\hat{\text{k}}}} \right){\text{E}}_{0} } \right]} \right\}{\text{e}}^{{ - {\text{j}}k( - \hat{r} + \hat{k}) \cdot {\text{r}}^{\prime } }} {\text{d}}S^{\prime } }$$

where ***k*** means wave vector, *λ* represents the wavelength in free space, *r* refers to the distance from the field point to the origin of coordinates. The integral term can be calculated separately, so there is the following expression:

5 $$   I = \iint\limits_{{S^{\prime } }} {{\hat{\text{r}}} \times \left\{ {{\hat{\text{r}}} \times \left[ {\left( {{\hat{\text{n}}}\left( {{\text{r}}^{\prime } } \right) \cdot {\text{E}}_{0} } \right){\hat{\text{k}}} - \left( {{\hat{\text{n}}}\left( {{\text{r}}^{\prime } } \right) \cdot {\hat{\text{k}}}} \right){\text{E}}_{0} } \right]} \right\}{\text{e}}^{{ - {\text{j}}k\left( { - \hat{r} + \hat{k}} \right) \cdot {\text{r}}^{\prime } }} {\text{d}}S^{\prime } } $$

Therefore, the RCS of the surface can be determined as

6 $$ \sigma_{{\text{PO}}} = \frac{{4\uppi }}{{\lambda^{2} }}\left| I \right|^{2} $$

**Figure 3:**
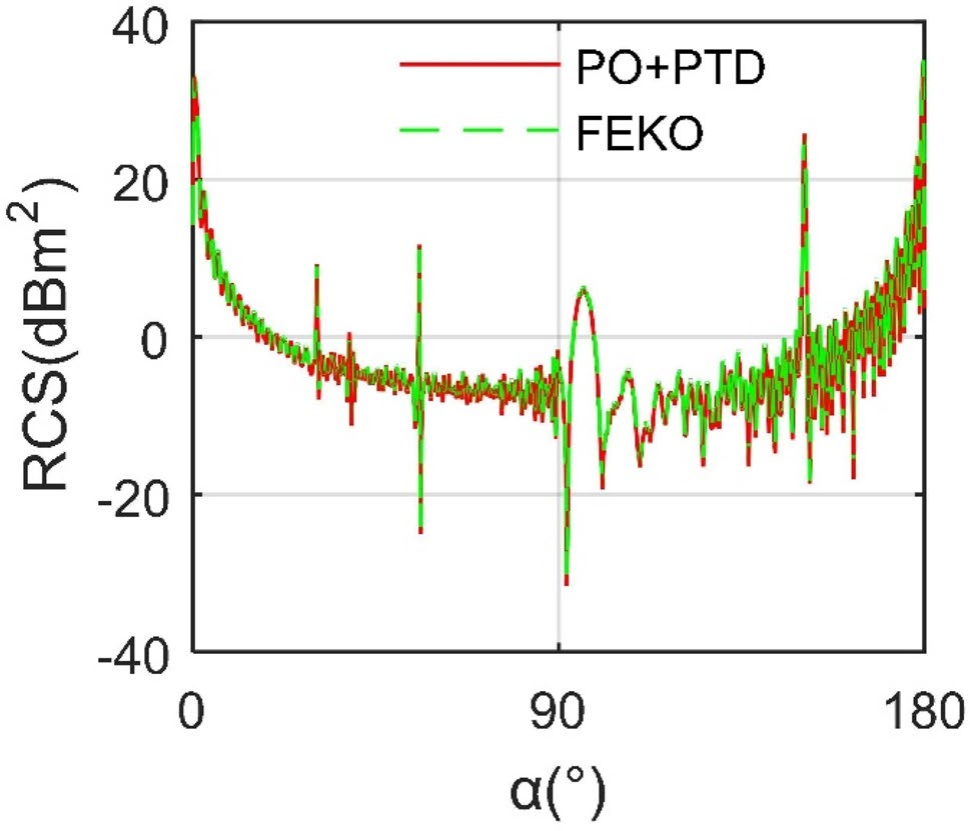
Verification of RCS calculation method on *m*_0_, *β* = 0°, *f*_R_ = 10 GHz.

In fact, the aircraft model has many edges and split angles, and these geometric features will also produce diffraction effects on electromagnetic waves. Here, PTD is used to solve the edge diffraction. ^20^ The total RCS can be expressed as the joint contribution of PO and PTD:

7 $$ \sigma { = }\left| {\sum\limits_{i = 1}^{{N_{F} }} {\left( {\sqrt {\sigma_{PO} } } \right)} \begin{array}{*{20}c} {} \\ i \\ \end{array} + \sum\limits_{j = 1}^{{N_{E} }} {\left( {\sqrt {\sigma_{PTD} } } \right)\begin{array}{*{20}c} {} \\ j \\ \end{array} } } \right|^{2} $$

where *N*_F_ refers to the number of facets and *N*_E_ represents the number of edges. The presented RCS calculation method is verified by PO + MOM (Method of Moment)/MLFMM (Multi-Level Fast Multipole Method) in FEKO (FEldberechnung bei Korpern mit beliebiger Oberflache) as shown in Fig. 3, where *f*_R_ is the radar wave frequency and the radar wave uses horizontal polarization. It can be seen that the two RCS curves are roughly coincident, and the result calculated by FEKO is slightly larger than the other, where the mean of the RCS curve of PO + PTD is smaller than that of FEKO by 0.3909 dBm^2^. This is because the two use different grid processing methods and different RCS algorithms. These results indicate that the RCS algorithm presented in this paper is accurate to deal with the electromagnetic scattering characteristics of the aircraft model.

### IR signature evaluation

Monte Carlo (MC) and ray tracing method (RTM) are used to solve the infrared signature of aircraft exhaust system^3^. Consider the absorption and emission in the RTE, without considering the scattering of the medium, so that the refractive index is always equal to 1, then RTE can be described as:

8 $$ \frac{{{\text{d}} L\left( {{\text{r}},{\text{s}}} \right)}}{{{\text{d}} s}} + aL\left( {{\text{r}},{\text{s}}} \right) = a\frac{{\sigma T^{4} }}{\uppi } $$

where ***r*** refers to the position vector, ***s*** is the direction vector, *s* represents the length along the path, *a* is the absorption coefficient, *L* is the radiance, *σ* refers to the Stefan-Boltzmann constant, *T* is the local temperature.

Using discrete coordinate model to calculate thermal radiation, RTE can be transformed into:

9 $$ \nabla \cdot \left[ {L\left( {{\text{r}},{\text{s}}} \right){\text{s}}} \right] + aL\left( {{\text{r}},{\text{s}}} \right) = a\frac{{\sigma T^{4} }}{\uppi } $$

According to MC + RTM, the spectral radiance reaching the receiving point of the detector can be expressed as:

10 $$ L_{\sigma } = L_{\sigma }^{0} \tau_{1\sigma } \tau_{2\sigma } \cdot \cdot \cdot \tau_{n\sigma } + L_{b\sigma }^{1} \left( {1 - \tau_{1\sigma } } \right)\tau_{2\sigma } \tau_{3\sigma } \cdot \cdot \cdot \tau_{n\sigma } + \cdot \cdot \cdot + L_{b\sigma }^{n - 1} \left( {1 - \tau_{{\left( {n - 1} \right)\sigma }} } \right)\tau_{n\sigma } + L_{b\sigma }^{n} \left( {1 - \tau_{n\sigma } } \right) $$

where $L_{\sigma }^{0}$ refers to spectral radiance of wall reverse rays, $L_{b\sigma }^{i}$ means spectral radiance, *τ*_*iσ*_ represents *i*-th layer spectral transmittance.

On this basis, the irradiance reaching the receiving point of the detector can be calculated as:

11 $$ E = \sum\limits_{i = 1}^{{N_{\text{b}} }} {\sum\limits_{j = 1}^{N} {L_{{\sigma ,\left( {i,j} \right)}}^{n} } } \cdot \cos \theta_{j} \cdot \Delta \Omega_{j} \cdot \Delta \sigma_{i} \cdot 100 $$

where *E* means the radiant illumination, *N*_b_ refers to the total number of wave bands, *N* represents the total number of rays contributing to the measurement point, *θ *_*j*_refers to the angle between the center of the *j*-th solid angle and the surface normal of its measuring point, ΔΩ _*j*_means the *j*-th solid angle, Δ*σ*_*i*_ is the width of the *i*-th wave band.

At this time, the radiation intensity can be determined as:

12 $$ I_{\sigma } = E_{\sigma } \left( R \right) \cdot R^{2} $$

where *I* is the radiation intensity and *R* refers to the linear distance between the aircraft and the IR detector.

For the infrared radiation of the exhaust pipe, regarding the exhaust pipe as a gray body, the total radiation and total radiation intensity can be expressed as:

13 $$ L_{\Delta \lambda } = \frac{\varepsilon }{\uppi }M_{{\lambda_{1} \sim \lambda_{2} }} = \frac{\varepsilon }{\uppi }\int_{{\lambda_{1} }}^{{\lambda_{2} }} {M_{\lambda } {\text{d}} \lambda } $$

14 $$ I_{\Delta \lambda } = L_{\Delta \lambda } \Delta A\cos \theta $$

where *λ* is the wavelength, $M_{{\lambda_{1} \sim \lambda_{2} }}$ represents the radiation emission degree of the black body between the bands *λ*_1_ ~ *λ*_2_. *ε* is the emissivity of the exhaust pipe, which is set to 0.9. Δ*A* is the area of the nozzle bin, *M*_*λ*_ means the spectral radiation emission degree of the black body.

Noting the Planck's formula:

15 $$ M_{\lambda } = \frac{{c_{1} }}{{\lambda^{5} }}\frac{1}{{e^{{c_{2} /\left( {\lambda T} \right)}} - 1}} $$

Thus the radiation emittance of blackbody in *λ*_1_ ~ *λ*_2_ band could be calculated as:

16 $$  M_{{\lambda _{1} \sim \lambda _{2} }}  = \frac{{15\sigma T^{4} }}{{\uppi ^{4} }}\int_{{c_{2} /\left( {\lambda _{2} T} \right)}}^{{c_{2} /\left( {\lambda _{1} T} \right)}} {\frac{{x^{3} }}{{e^{x}  - 1}}} {\text{d}}x  $$

**Figure 4:**
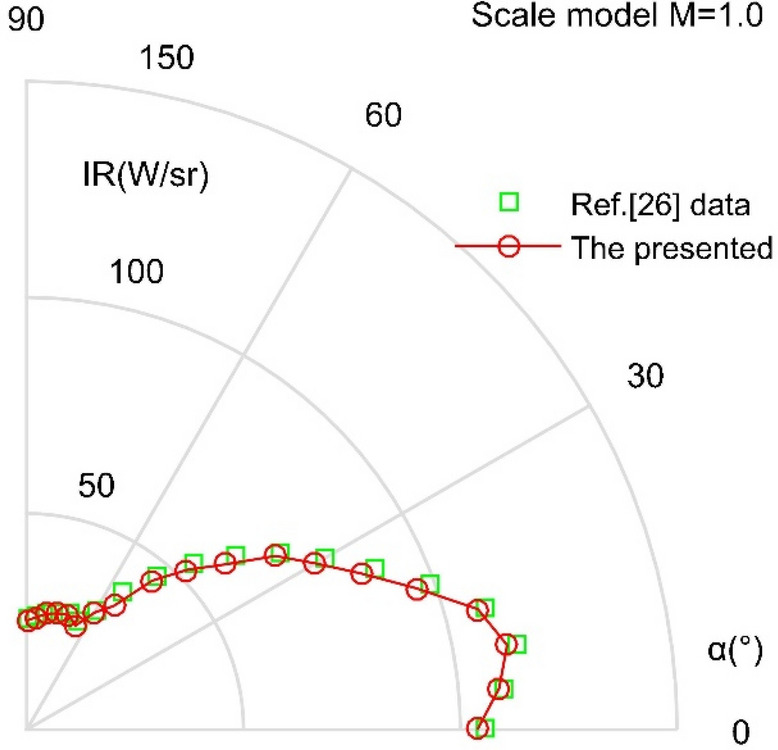
Verification of IR signature calculation method, 3–5 μm wavelength band.

The radiation constants have the following relationship:

17 $$ \begin{array}{*{20}c} {c_{1} = 2\uppi hc^{2} ,} & {c_{2} = \frac{hc}{k},} & {\sigma = \frac{{2\uppi ^{5} k^{4} }}{{15c^{2} h^{3} }}} \\ \end{array} $$

where *h* refers to the Planck constant, *h* = 6.626176 × 10^-34^ J·s, *k* = 1.38 × 10^-23^ J/K, *c* is the speed of light. The IR signature calculation method presented here is verified as shown in Fig. 4, where the calculation results presented here are basically consistent with the known data in the literature^26^. The mean of the red data is 1.67 W/sr smaller than that of the green data. These results indicate that the IR calculations in this paper are feasible and accurate for evaluating the infrared radiation characteristics of this aircraft exhaust system.

### Comprehensive stealth design

In order to improve the radar/infrared stealth performance of this aircraft, the objective function is defined as follows:

18 $$ \left\{ {\begin{array}{*{20}l} {\min :f_{{i_{{\text{ind}}} }} \left( m \right)\begin{array}{*{20}c} , & i \\ \end{array}_{{{\text{ind}}}} = 1,2,3} \hfill \\ {M = \left\{ {m_{1} ,m_{2} ,...,m_{n} } \right\}} \hfill \\ \end{array} } \right. $$

where *i*_ind_ is the serial number, *m* is the individual of the aircraft model, *M* is the collection of descendants^20,25^, noting that this expression implies that the objective function pursues the minimum of these three indicators. The comprehensive stealth indicators are:

19 $$ \left\{ {\begin{array}{*{20}l} {f_{1} \left( m \right) = \sigma_{\text{m}} } \hfill \\ {f_{2} \left( m \right) = \sigma_{t} } \hfill \\ {f_{3} \left( m \right) = I_{{\text{n}}} } \hfill \\ \end{array} } \right.\begin{array}{*{20}c} {} & {\left| {\begin{array}{*{20}l} {0^{ \circ } \le \alpha \le 180^{ \circ } \begin{array}{*{20}c} , & {\beta = 0^{ \circ } } \\ \end{array} } \hfill \\ {\begin{array}{*{20}c} {f_{\text{R}} = 10{\text{GHz}} ,} & {{\text{HH}}} \\ \end{array} } \hfill \\ { - 30^{ \circ } \le \alpha_{n} \le 30^{ \circ } } \hfill \\ \end{array} } \right.} \\ \end{array} $$

where HH represents that the radar wave is horizontally polarized, *α*_*n*_ means the observation angle in the normal observation field, *σ*_m_ is the mean RCS indicator, *σ*_t_ is the tail RCS indicator, *I*_n_ is the infrared radiation intensity indicator in the normal observation field.

Throughout the overall design process, equation constraints are expressed as follows:

20 $$ \left\{ {\begin{array}{*{20}l} {W_{{\text{f}}} \left( {M_{{i_{\text{e}} }} } \right) - W_{{\text{f}}} \left( {M_{{j_{\text{e}} }} } \right) = 0} \hfill \\ {L_{\text{e}} \left( {M_{{i_{\text{e}} }} } \right) - L_{\text{e}} \left( {M_{{j_{\text{e}} }} } \right) = 0} \hfill \\ {X_{\text{n}} \left( {M_{{i_{\text{e}} }} } \right) - X_{\text{n}} \left( {M_{{j_{\text{e}} }} } \right) = 0} \hfill \\ \end{array} } \right.\begin{array}{*{20}c} {\begin{array}{*{20}c} {} & \& \\ \end{array} } & {\left\{ {\begin{array}{*{20}l} {D_{{\text{in}}} \left( {M_{{i_{\text{e}} }} } \right) - D_{{\text{in}}} \left( {M_{{j_{\text{e}} }} } \right) = 0} \hfill \\ {L_{{\text{in}}} \left( {M_{{i_{\text{e}} }} } \right) - L_{{\text{in}}} \left( {M_{{j_{\text{e}} }} } \right) = 0} \hfill \\ {X_{in} \left( {M_{{i_{\text{e}} }} } \right) - X_{in} \left( {M_{{j_{\text{e}} }} } \right) = 0} \hfill \\ \end{array} } \right.} \\ \end{array} \begin{array}{*{20}c} {\begin{array}{*{20}c} {} & {\left| {\begin{array}{*{20}c} {} \\ {} \\ {\forall i_{{\text{e}}} ,j_{\text{e}} \in \left\{ {1,2,...,k} \right\}} \\ \end{array} } \right.} \\ \end{array} } & {} \\ \end{array} $$

where *W*_f_ is the width of the aircraft fuselage, *D*_in_ is the diameter of the end of the intake pipe, *L*_e_ is the length of the engine compartment, *L*_in_ represents the length of the air intake, *X*_n_ means the *x* coordinate of the front face of the nozzle, *X*_in_ refers to the *x* coordinate of the end face of the intake pipe, and *k* is the number of generations.

The inequality equality constraints can be expressed as:

21 $$ \left\{ {\begin{array}{*{20}l} {L_{\text{f}} \left( {M_{{i_{{{\text{ie}}}} }} } \right) - L_{f,max} \le 0} \hfill \\ {L_{ex} \left( {M_{{i_{{{\text{ie}}}} }} } \right) - L_{ex,\max } \le 0} \hfill \\ \end{array} } \right.\begin{array}{*{20}c} {} & \& \\ \end{array} \left\{ {\begin{array}{*{20}l} {D_{x} \left( {M_{{j_{{\text{ie}}} + 1}} } \right) - D_{x} \left( {M_{{j_{{\text{ie}}} }} } \right) \le 0} \hfill \\ {T_{{\text{n}}} \left( {M_{{j_{{\text{ie}}} + 1}} } \right) - T_{\text{n}} \left( {M_{{j_{{\text{ie}}} }} } \right) \le 0} \hfill \\ \end{array} } \right.\left| {\begin{array}{*{20}l} {\forall i_{{{\text{ie}}}} \in \left\{ {1,2,...,k} \right\}} \hfill \\ {\forall j_{{\text{ie}}} \in \left\{ {1,2,...,k - 1} \right\}} \hfill \\ \end{array} } \right. $$

where *L*_f_ refers to the length of the fuslage, *L*_ex_ is the length of the exhaust pipe, *L*_f,max_, and *L*_ex,max_ are the limit values, *D*_x_ is the drag indicator, *T*_n_ is the temperature indicator of nozzle face.

When there are optimal solutions for the stealth indicators, the following relationship (COM test^20,25^) should be satisfied:

22 $$ \left\{ {\begin{array}{*{20}l} {f_{{i_{{{\text{ind}}}} }} \left( M \right) \ge f_{{i_{{{\text{ind}}}} }} \left( {M^{*} } \right)} \hfill \\ {D_{x} \left( {M^{*}_{{j_{{\text{ie}}} + 1}} } \right) \le D_{x} \left( {M_{{j_{{\text{ie}}} }} } \right)} \hfill \\ {T_{n} \left( {M^{*}_{{j_{{\text{ie}}} + 1}} } \right) \le T_{n} \left( {M_{{j_{{\text{ie}}} }} } \right)} \hfill \\ \end{array} } \right.\left| {\begin{array}{*{20}l} {} \hfill \\ {\forall i_{{{\text{ind}}}} \in \left\{ {1,2,3} \right\}} \hfill \\ {\forall j_{{\text{ie}}} \in \left\{ {1,2,...,k - 1} \right\}} \hfill \\ \end{array} } \right. $$

When the optimal solution does not exist, that is, these stealth indicators cannot reach the minimum at the same time, then the Pareto solution needs to be applied:

23 $$ \left\{ {\begin{array}{*{20}l} {f_{{i_{{{\text{ind}}}} }} \left( {M^{*} } \right) \le f_{{i_{{{\text{ind}}}} }} \left( M \right)} \hfill \\ {D_{x} \left( {M_{{j_{{\text{ie}}} }} } \right) \ge D_{x} \left( {M^{*}_{{j_{{\text{ie}}} + 1}} } \right)} \hfill \\ {T_{n} \left( {M_{{j_{{\text{ie}}} }} } \right) \ge T_{n} \left( {M^{*}_{{j_{{\text{ie}}} + 1}} } \right)} \hfill \\ \end{array} } \right.\left| {\begin{array}{*{20}l} {} \hfill \\ {\exists i_{{{\text{ind}}}} \in \left\{ {1,2,3} \right\}} \hfill \\ {\exists j_{{\text{ie}}} \in \left\{ {1,2,...,k - 1} \right\}} \hfill \\ \end{array} } \right. $$

In order to establish individuals with better stealth performance, a ranking factor can be defined here to distinguish these feasible solutions in each generation:

24 $$ f_{\text{s}} \left( {j_{{\text{ie}}} ,i_{{\text{ind}}} } \right) = r_{{\text{ind}}} \left( {j_{{\text{ie}}} ,i_{{\text{ind}}} } \right)/N_{ind} $$

25 $$ f_{r} \left( {j_{{\text{ie}}} } \right) = \sum\limits_{j = 1}^{{N_{indc} }} {f_{\text{s}} \left( {j_{{\text{ie}}} ,i_{{\text{ind}}} } \right)} /N_{indc} $$

where *f*_s_(*j*_ie_, *i*_ind_) represents the sorting factor of the *i*_ind_-th stealth indicator of the *j*_ie_-th individual, *N*_ind_ is the number of individuals in the current offspring, *r*_ind_(*j*_ie_, *i*_ind_) indicates the ascending ranking of the *i*_ind_ -th indicator of the *j*_ie_ -th individual, *f*_r_ is the ranking factor, *N*_indc_ is the number of stealth indicators.

At this time, the optimal solution can be expressed as the individual with the smallest *f*_r_:

26 $$ M* = m_{{j_{{\text{ie}}} }} \left| {\min \left( {f_{\text{r}} \left( {j_{{\text{ie}}} } \right)} \right)} \right. $$

If the above ranking factor Pareto section is still not unique, the priority is defined to continue to distinguish these individuals:

27 $$ p_{{\text{indc}}} \left( {\sigma_{\text{m}} } \right) \ge p_{{\text{indc}}} \left( {\sigma_{\text{t}} } \right) \ge p_{{\text{indc}}} \left( {I_{n} } \right) $$

where *p*_indc_ is the priority of individual stealth indicator. At this time, priority is given to the individual whose RCS mean indicator performs better.

## Model

**Figure 5:**
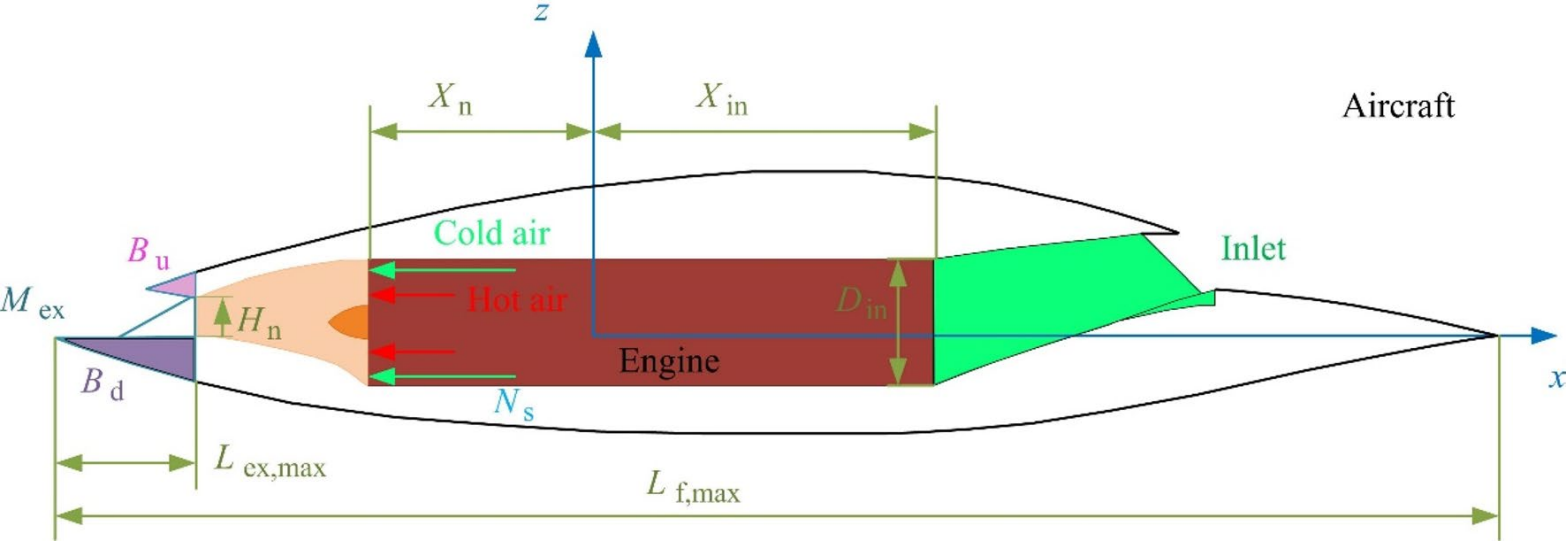
Aircraft design draft and parameter variables, drawn using Microsoft Visio 2007.

The outline design of the aircraft symmetry plane is shown in Fig. 5, where *M*_ex_ represents the model of the exhaust port, *B*_d_ is the nozzle model with downward blocking, *N*_s_ refers to the number of nozzle stages, *B*_u_ is a nozzle model with upward blocking, *H*_n_ is the height of the nozzle.

**Figure 6:**
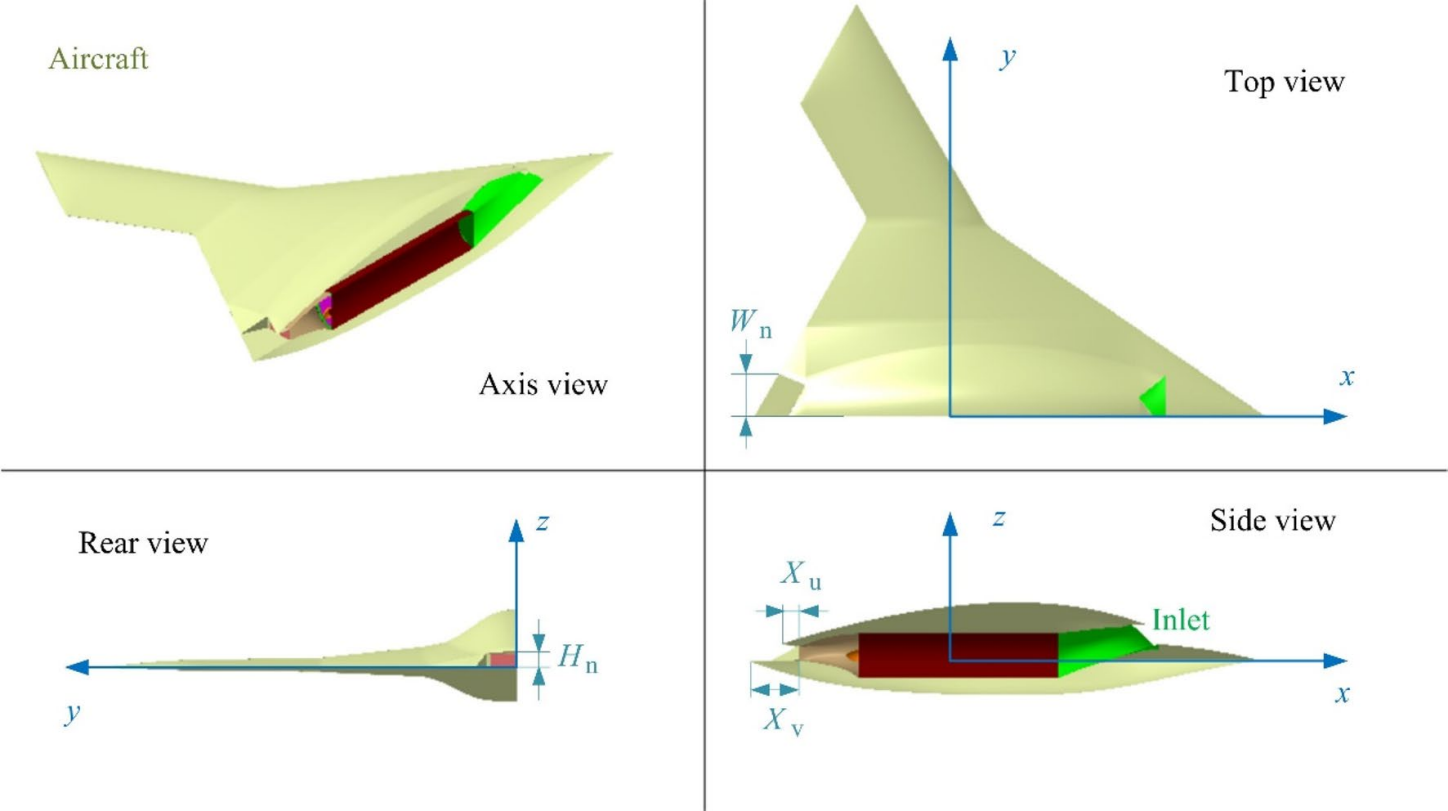
Three-view display of aircraft geometric model, drawn using Microsoft Visio 2007 and CATIA V5R20.

The model of the unmanned aircraft was built as shown in Fig. 6, where *W*_n_ is a custom parameter used to determine the nozzle width, *X*_v_ is a custom length parameter used to determine the size of *B*_d_, noting that the outer edge of *B*_d_ is parallel to the tail edge of the aircraft. *X*_u_ is the size parameter of *B*_u_. The main geometric size data of *m*_0_ is shown in Table 1, where *H*_f_ is the height of the fuselage, *W*_b_ is wingspan, *A*_sl_ is the sweep angle of wing leading edge, *A*_sn_ is the sweep angle of nose, *A*_ft_ is forward sweep angle of the trailing edge of the fuselage. When the parameters of the exhaust system change, the aircraft model also changes, and the aircraft model here can be represented by the following parameters or sub-models:

28 $$ m = \left\{ {M_{{\text{ex}}} ,N_{\text{s}} ,B_{\text{d}} ,H_{\text{n}} ,W_{\text{n}} ,B_{\text{u}} } \right\} $$

**Table 1:** The main geometric size data of *m*_0_.

Parameter	*L*_f_ (m)	*X*_n_ (m)	*A*_sl_ (°)	*H*_f_ (m)	*W*_b_ (m)
Value	11.041	-2	30.518	2.082	18.5
Parameter	*W*_n_ (m)	*D*_in_ (m)	*X*_in_ (m)	*A*_sn_ (°)	*A*_ft_ (°)
Value	0.8	0.5	2.5	55.717	29.938

where the parameters in Eq. (28) are set as variable parameters, the parameters in Eq. (20) are restricted to constant parameters.

**Figure 7:**
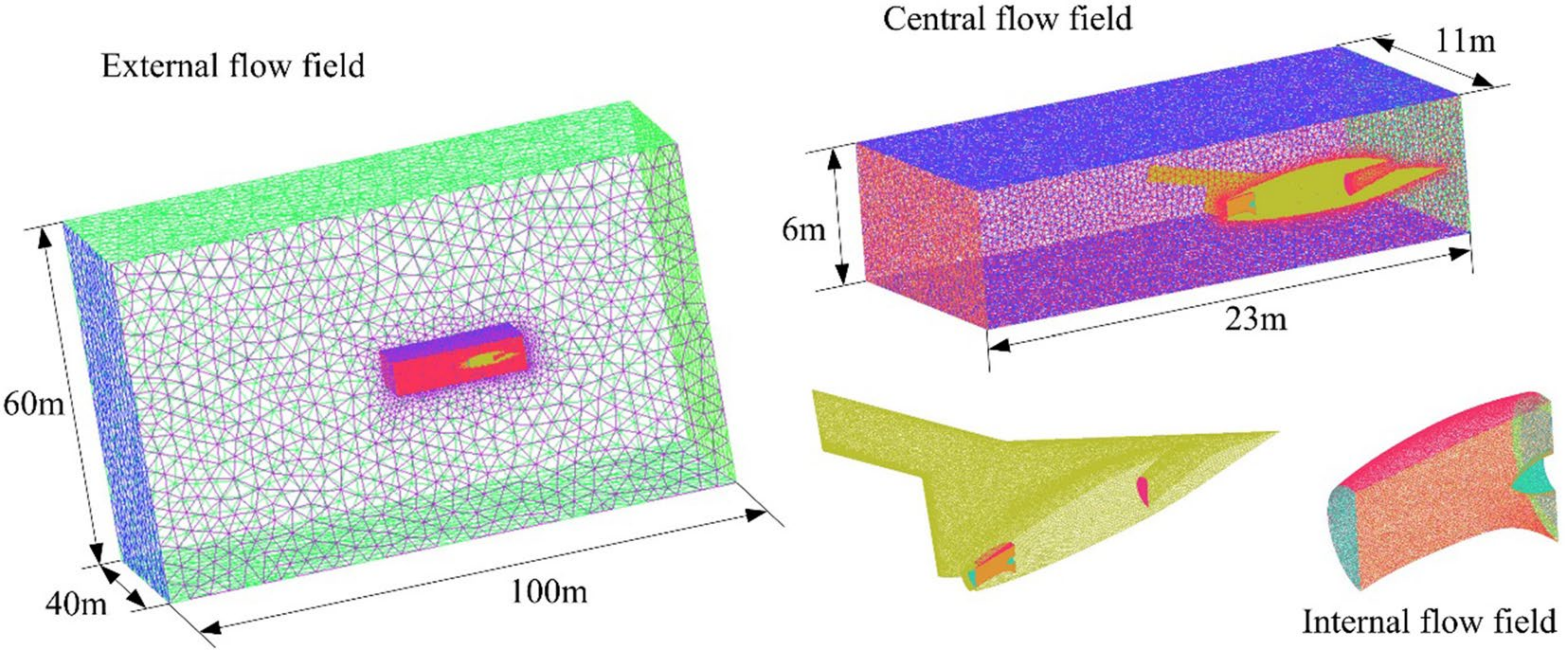
Aircraft exhaust system flow field construction and grid division, drawn using Microsoft Visio 2007 and ANSYS ICEM 16.0.

The flow field of the aircraft exhaust system is established as shown in Fig. 7, where high-precision unstructured grid technology is used to divide the external, central and internal flow field areas. Mesh encryption is added to surfaces and edges with small dimensions or large curvature changes, including air intakes, exhaust ports, center body, nozzle pipe, leading and trailing edges of wings. The velocity of the incoming flow in the far front of the aircraft is set to 85 m/s with the temperature 300 K, while the high-temperature airflow at the nozzle inlet is set at 870 K. The standard *k*-*ε* model is used to solve the Navier-Stocks equation of the exhaust system flow field. Momentum, density, turbulent kinetic energy, energy and turbulent dissipation rate are discretized using a second-order upwind scheme.

**Figure 8:**
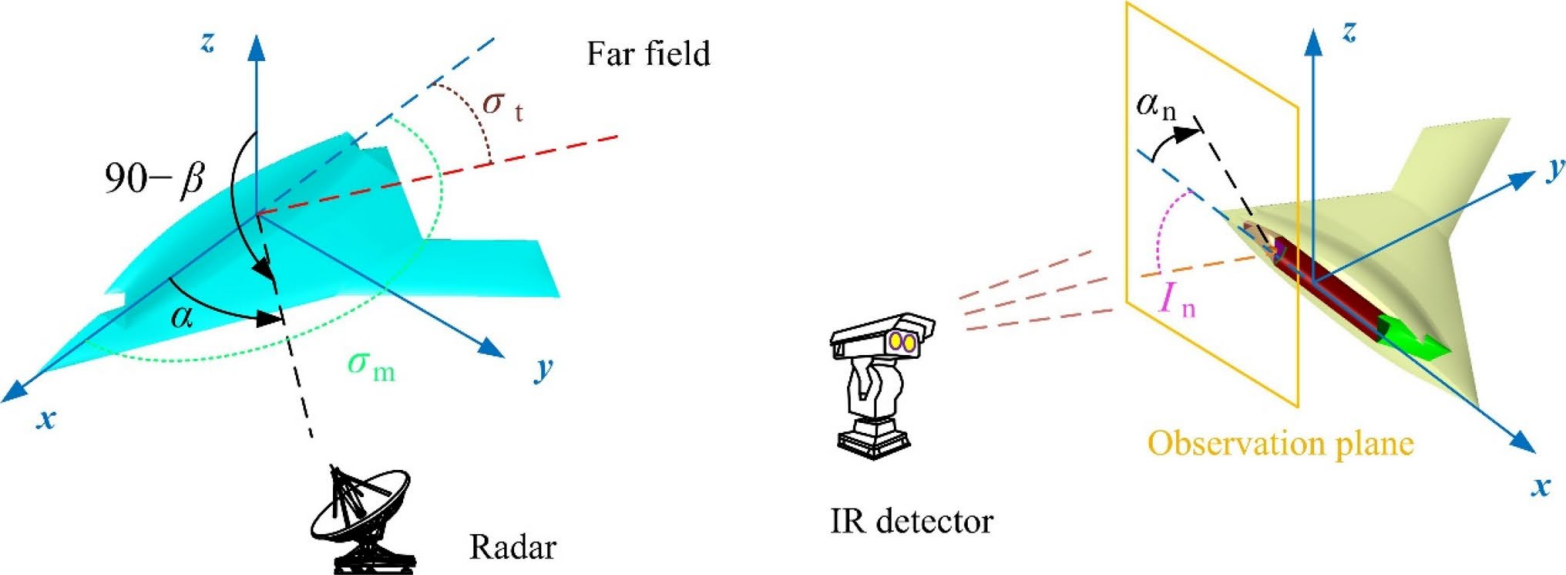
Observation field setting of aircraft radar cross section and IR signature, *β* = 0°, drawn using Microsoft Visio 2007 and CATIA V5R20.

In order to evaluate the changes in stealth characteristics brought to the aircraft during the design of the exhaust system, the radar and infrared observation fields are set as shown in Fig. 8, where *σ*_m_ is equal to the mean RCS in the range of 0° ≤ *α* ≤ 180°, *σ*_t_ is equal to mean RCS in the range of 150° ≤ *α* ≤ 180°, *I*_n_ is equal to the mean value of the IR radiation intensity in the range of –30° ≤ *α*_n_ ≤ 30°.

## Results and discussion

**Figure 9:**
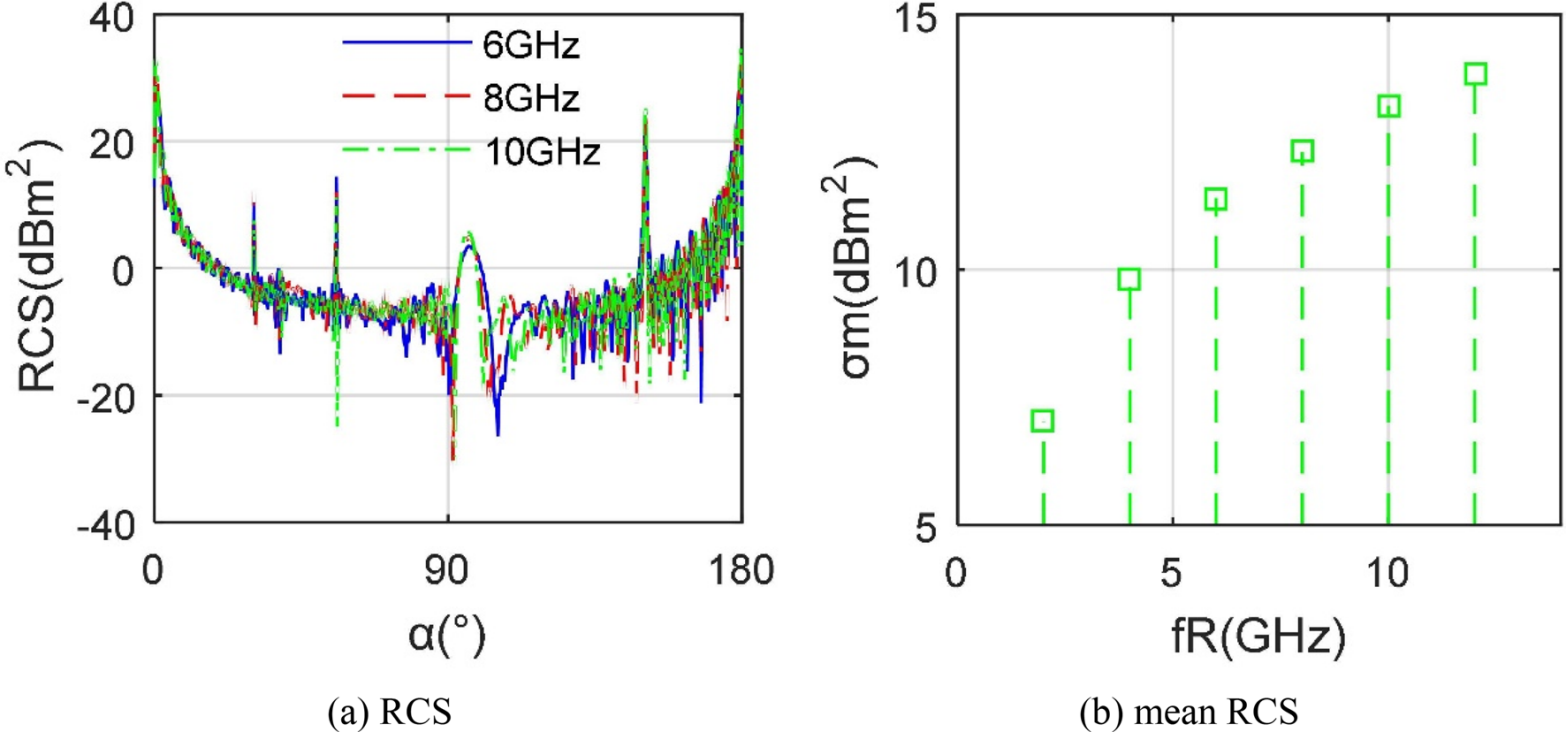
RCS of aircraft model under different radar wave frequencies, *β* = 0°.

Figure 9 presents that the aircraft's RCS and RCS mean indicators show great differences under different radar wave frequencies. When *f*_R_ is increased from 6 to 10 GHz, the RCS curve is generally consistent, but the local amplitude slightly increases, which is very obvious in the peaks of the head, side and tail. The increasing speed of the RCS mean index gradually decreases with the increase of the radar wave frequency. The *σ*_m_ at 2 GHz is only around 7 dBm^2^, and the index at 12 GHz reaches 13.8551 dBm^2^. In order to make the calculation results of the electromagnetic scattering level of the aircraft comparable in the design process, the radar wave frequency here is set to 10 GHz.

### Effects of M_ex_ and N_s_

**Figure 10:**
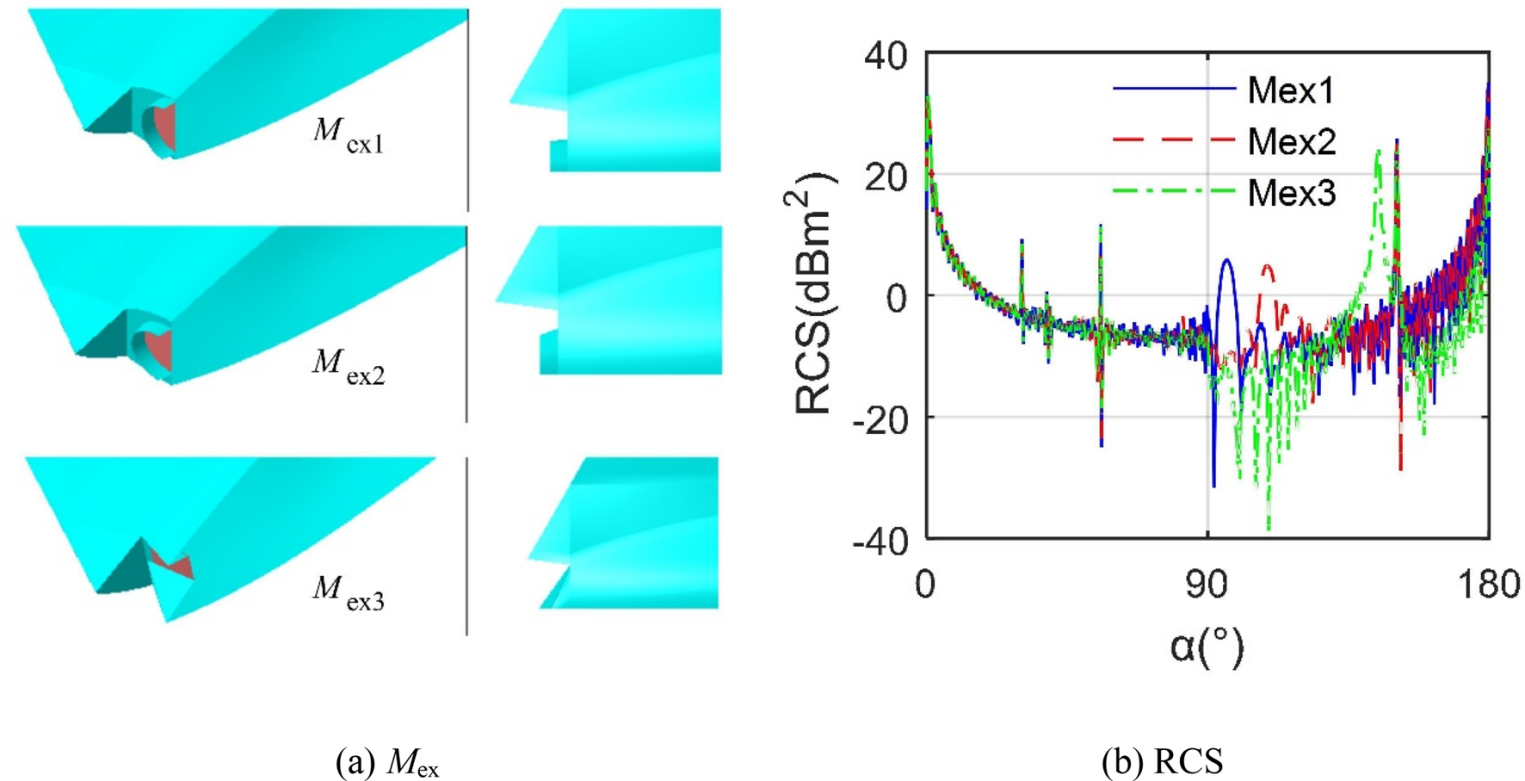
RCS of aircraft under different exhaust port models, *f*_R_ = 10 GHz, *β* = 0°, drawn using Microsoft Visio 2007 and CATIA V5R20.

Figure 10 provides that these three exhaust port models have a great influence on the RCS of the aircraft, where *M*_ex1_ uses a simple round pipe, *M*_ex2_ uses an oval design, and *M*_ex3_ uses a rectangular nozzle with a triangular upper and lower baffle. It can be seen that the RCS level of *M*_ex3_ is lower than the other two, but it produces a peak of 24.41 dBm^2^ at 144.5° because *M*_ex3_ does not use the arc design of the first two, but accepts a more concise triangle + large acute angle nozzle, this measure can effectively deflect most radar waves to non-threatening azimuth. The RCS curves of *M*_ex1_ and *M*_ex2_ are generally similar, but the peaks and fluctuations in the lateral 92.25° ~ 116.5° are different, where the average RCS of *M*_ex2_ curve is 13.1265 dBm^2^, and that of *M*_ex3_ is 11.6694 dBm^2^. These results show that the reasonable design of the nozzle can effectively reduce the average RCS of the aircraft and the peak RCS of certain azimuths.

**Figure 11:**
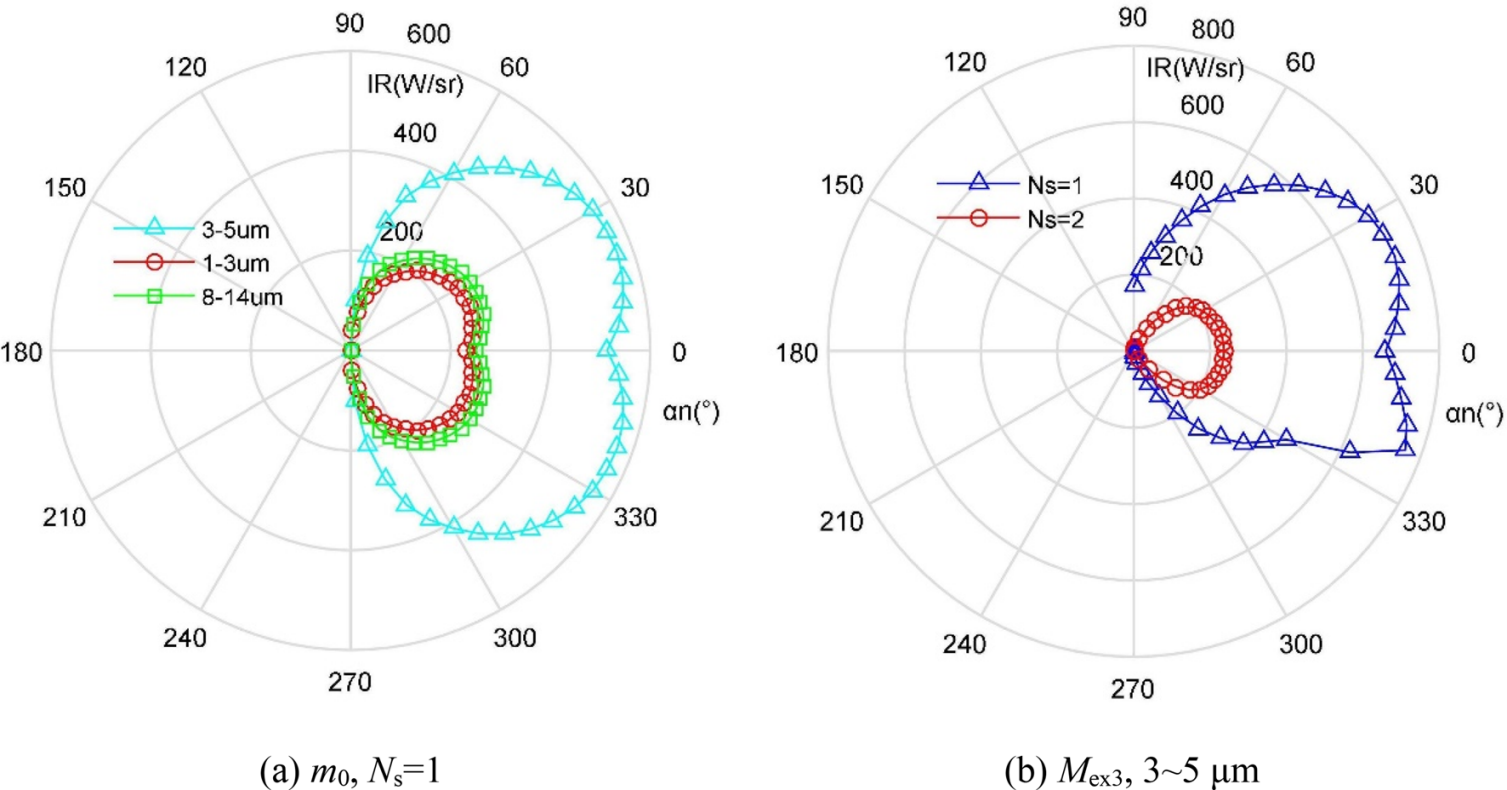
Aircraft IR signature under different wave bands and *N*_s_.

Figure 11 shows that the IR signatures of aircraft in different bands are very different, and the radiation intensity in the 3 ~ 5 μm band is significantly higher than the other two bands. In order to make the infrared radiation index results in the entire comprehensive design process comparable, the following IR calculations are performed in the 3 ~ 5 μm band. For the *M*_ex3_, the IR value in the positive observation angle range is significantly larger than that in the negative observation angle range, because the size of the upper baffle of the nozzle is smaller than that of the lower baffle, and the effect of blocking the heat source is small. The maximum IR value when *N*_s_ = 1 reaches 756.41 W/sr, while the IR curve at *N*_s_ = 2 as a whole is much smaller than the IR curve at *N*_s_ = 1, because at this time the introduction of circular cold air can better surround the high-temperature heat flow and form a certain isolation and protection, these combined effects greatly reduce the IR performance. These results show that the nozzle based on the principle of two-stage ejection can significantly improve the infrared radiation characteristics of the aircraft.

### Effects of B_d_ and H_n_

**Figure 12:**
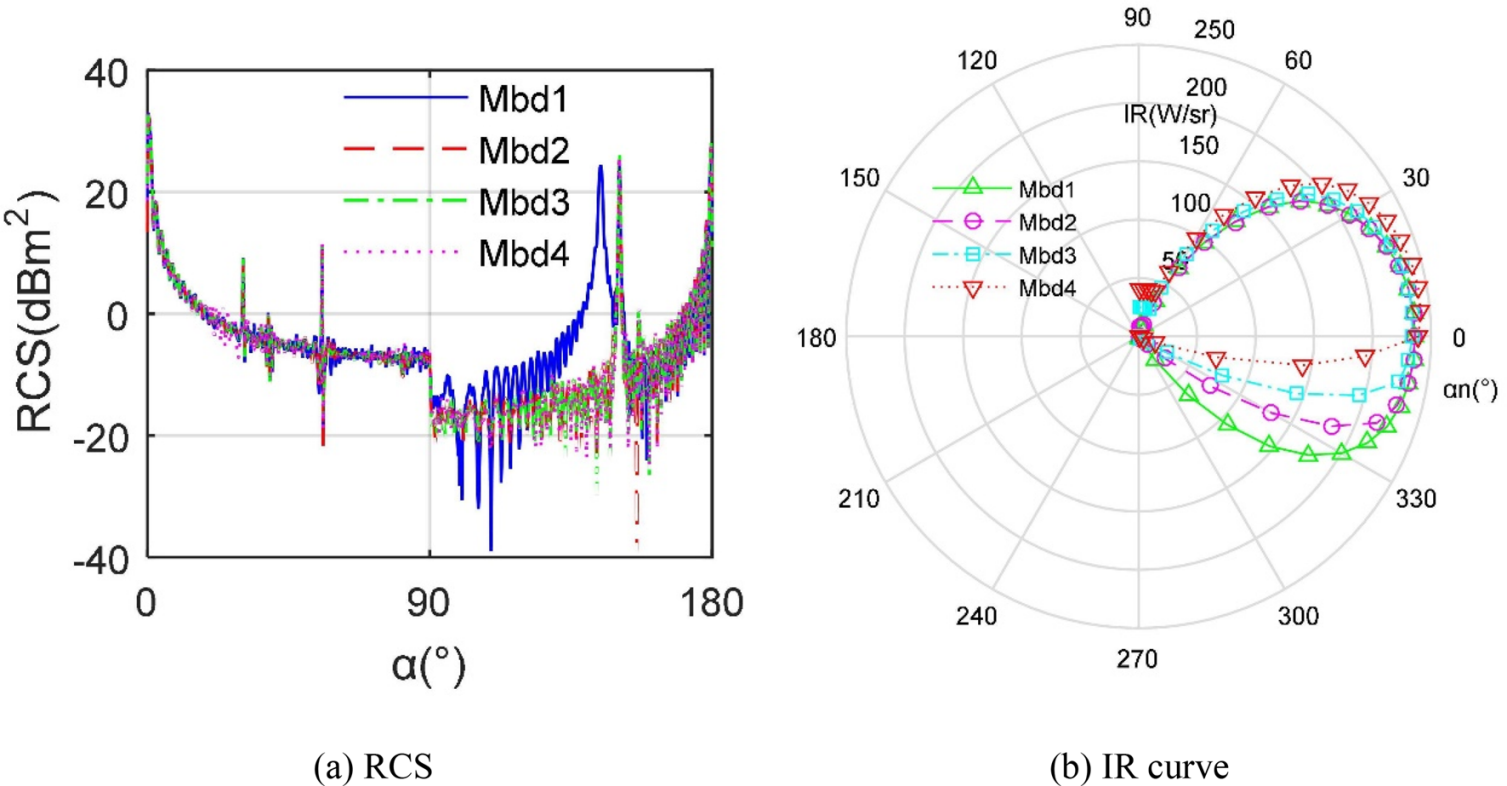
Comparison of stealth characteristics of aircraft under different *M*_bd_ models.

Figure 12 provides that the effect of different *M*_bd_ models on the stealth performance of the aircraft is obvious. As the value of *X*_v_ increases, the size of the lower baffle of the nozzle becomes larger and gradually integrates with the aircraft. This design results in a more concise shape of the entire aircraft and a stable deflection effect on electromagnetic waves, thus the RCS curves of *M*_bd2_, *M*_bd3_, and *M*_bd4_ are very similar. The RCS curve of *M*_bd1_ has a peak of 24.41 dBm^2^ at 144.5°, and the last three RCS curves have peaks at 150.5°. For the IR curves, the IR curve clearly shows an upward contraction trend with the increase of *X*_v_, this is because the outward extension of the lower baffle effectively blocks the radiation characteristics in the negative observation angle. As shown in Table 2, the difference between the radar stealth indicators under these four *M*_bd_ models is relatively small, but the IR indicator has a significant decreasing trend with a reduction of 65.5709 W/sr. These results indicate that the design of *M*_bd_ contributes more to the infrared stealth of the aircraft.

**Table 2:** Comparison of stealth indicators of aircraft under different *M*_bd_ models.

*M*_bd_	1	2	3	4
*X*_v_ (mm)	500	650	850	1150
*σ*_m_ (dBm^2^)	11.6694	11.0772	11.1147	11.0857
*σ*_t_ (dBm^2^)	12.3457	12.6437	12.5575	12.5467
*I*_n_ (W/sr)	223.7332	212.3998	187.0830	158.1623

**Figure 13:**
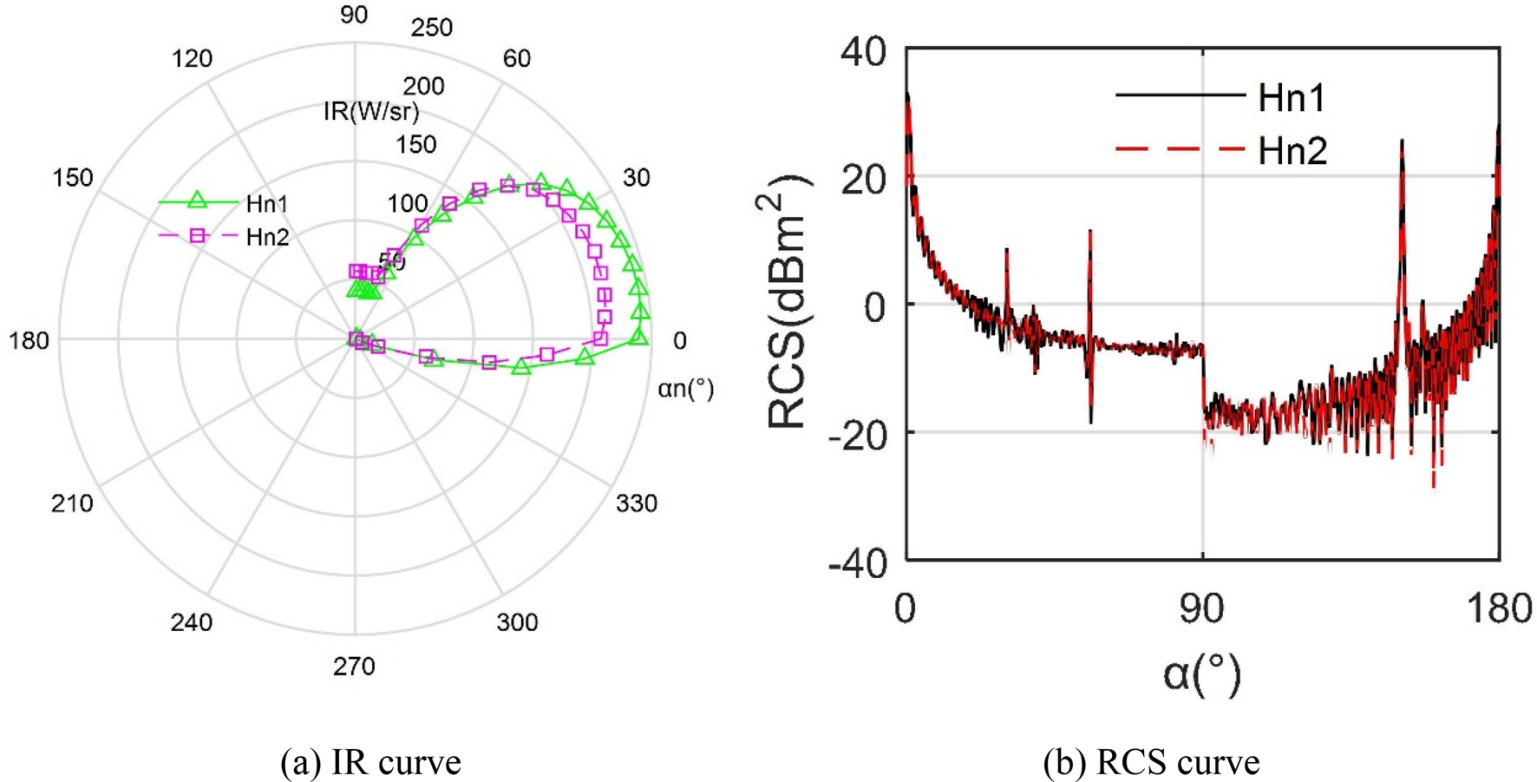
Effect of exhaust port height on aircraft stealth performance.

Figure 13 indicates that the height of the exhaust port will also have a more obvious impact on the stealth performance of the aircraft, where *H*_n1_ = 330 mm, *H*_n2_ = 300 mm. For the IR curves, the IR value of *H*_n1_ is obviously greater than the value of *H*_n2_ except the observation angle is greater than 45°, because reducing the height of the nozzle can increase the mixing intensity of the hot and cold air flow, which results in the normal infrared radiation effect of the nozzle being suppressed, but the blocking effect of the upper baffle on the nozzle is slightly weakened, thus the infrared radiation of the nozzle didn't weaken much when the observation angle increases positively. Note that the IR indicator at *H*_n1_ is around 158 W/sr, while that at *H*_n2_ is reduced to 140.0023 W/sr. The RCS curves under the two *H*_n_ are very similar, where the mean indicator here differs by only 0.1285 dBm^2^. These results indicate that choosing a suitable nozzle height is beneficial to improve the radar/infrared performance of the aircraft.

### Effects of W_n_ and B_u_

**Figure 14:**
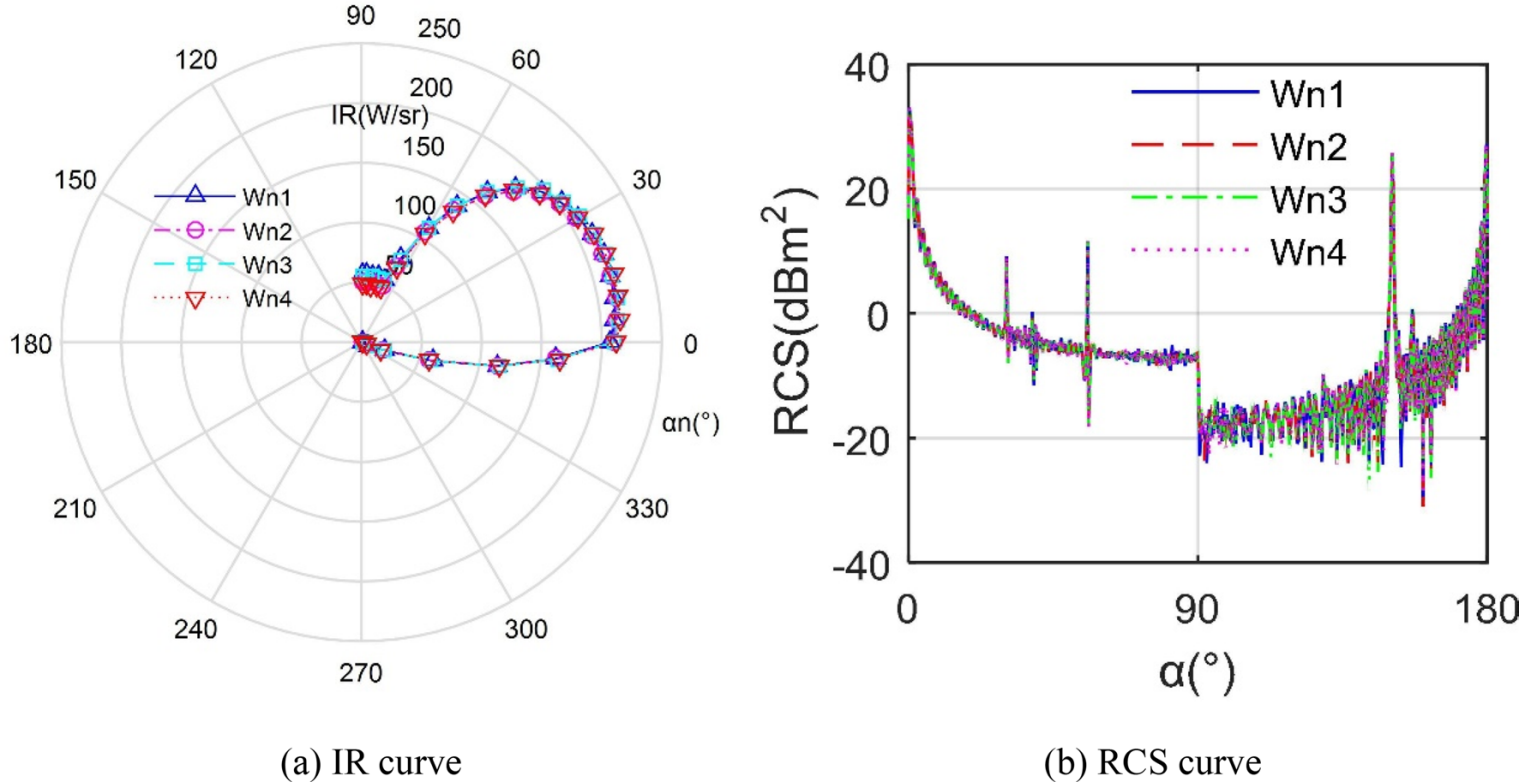
Comparison of stealth characteristics of aircraft under different *W*_n_.

Figure 14 manifests that the difference between RCS and IR curves of aircraft under different *W*_n_ is small. For the IR curves, the values of the four curves are relatively large in the range of 0° ~ 35°, exceeding 165.7 W/sr. At this time, the IR of *W*_n1_ and *W*_n2_ is slightly smaller than the other two. Overall, the IR radiation characteristics at these four *W*_n_ values are similar, because the opening width of the nozzle is defined on the edge of the tail of the aircraft. For the heat flow that is about to leave the tail of the aircraft, the mixing effect brought by *W*_n_ will have little effect on the wall near the outer baffle. For the RCS results, the four curves are very similar, including shape, maximum value, peak size, peak position and number of fluctuations. In the range of 90.75° ~ 108.8°, the difference between the four curves is somewhat obvious, because the increase of *W*_n_ reduces the surface area and average height of the side baffle outside the spout, which brings changes to the lateral electromagnetic scattering characteristics of the aircraft. As shown in Table 3, the results of the various stealth indicators under different *W*_n_ values are very similar, where the maximum difference of the RCS tail indicator is only 0.051 dBm^2^. These also show that the impact of *W*_n_ on the overall stealth performance of the aircraft is very limited within the given range.

**Table 3:** Comparison of stealth indicators of aircraft under different *W*_n_.

*W*_n_ number	1	2	3	4
*W*_n_ (mm)	800	850	900	950
*σ*_m_ (dBm^2^)	10.9572	10.9806	10.9583	10.9544
*σ*_t_ (dBm^2^)	12.0435	12.0527	12.0426	12.0017
*I*_n_ (W/sr)	140.0023	139.8559	141.4906	141.2781

**Figure 15:**
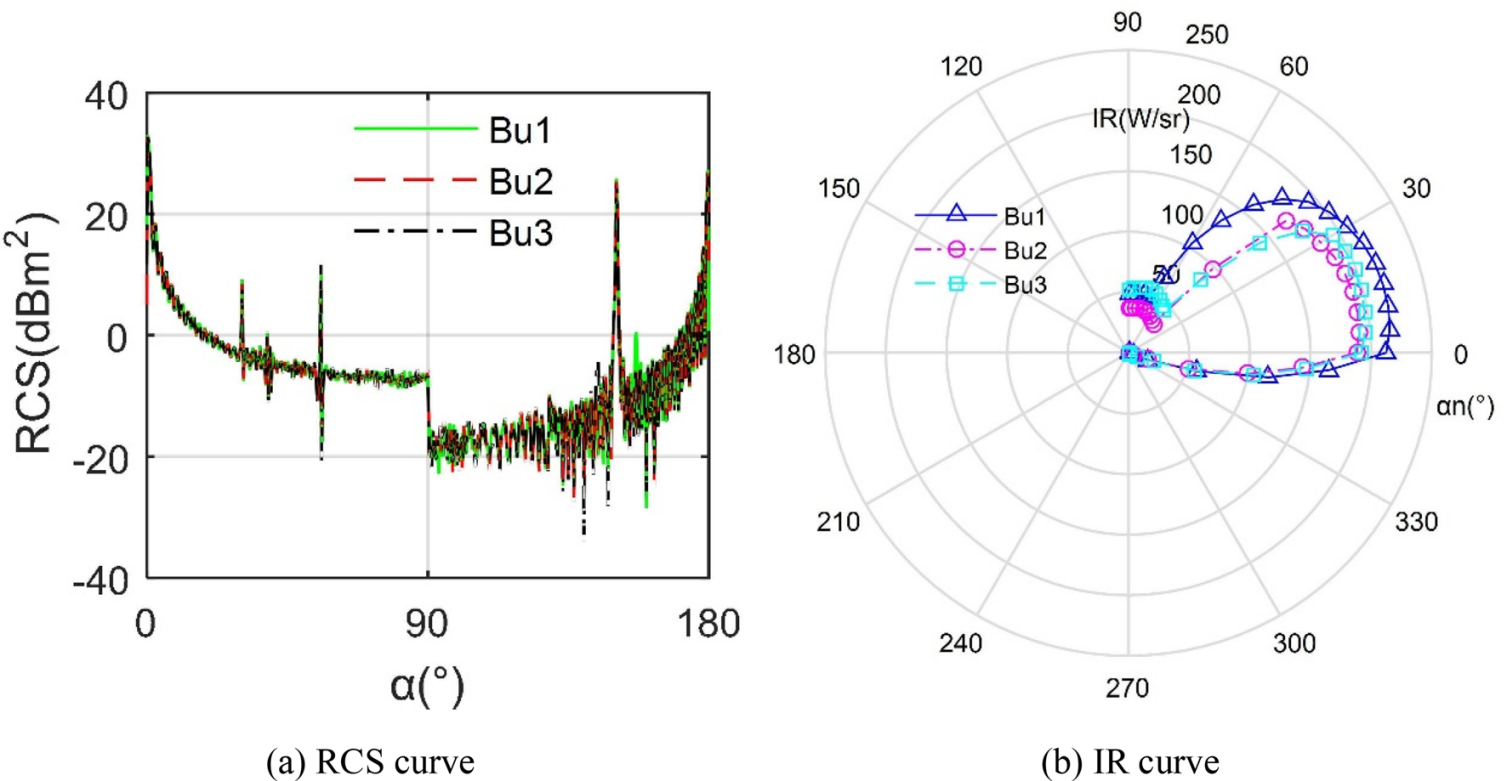
Radar/infrared stealth characteristics of aircraft under different *B*_u_.

Figure 15 investigates that different *B*_u_ parameters will affect the radar and infrared stealth characteristics of the aircraft, and the impact on the latter is more obvious, where *X*_u1_ = 300 mm, *X*_u2_ = 400 mm and *X*_u3_ = 500 mm. For the RCS results, the three curves are very similar in shape, and all produce large peaks at 0.25°, 150.5° and 179.8° with the maximum peak reaching 33.04 dBm^2^. For infrared radiation characteristics, the IR curve of *B*_u1_ is obviously larger than that of the other two. The IR values of *B*_u1_ and *B*_u2_ are almost equal in the negative observation angle range, and the IR result of *B*_u3_ in the positive observation angle range is larger than that of *B*_u2_, because the size of the upper baffle outside the nozzle increases and extends outward with the increase of *X*_u_, which is helpful to induce the airflow at the back of the fuselage to flow behind the center of the tail flame. This is conducive to the mixing of hot and cold airflow to a certain extent, but it also affects the radiation angle of the tail nozzle. These results indicate that *B*_u_ is a non-negligible factor affecting the performance of aircraft radar and infrared stealth performance.

### Comprehensive results discussion

**Figure 16:**
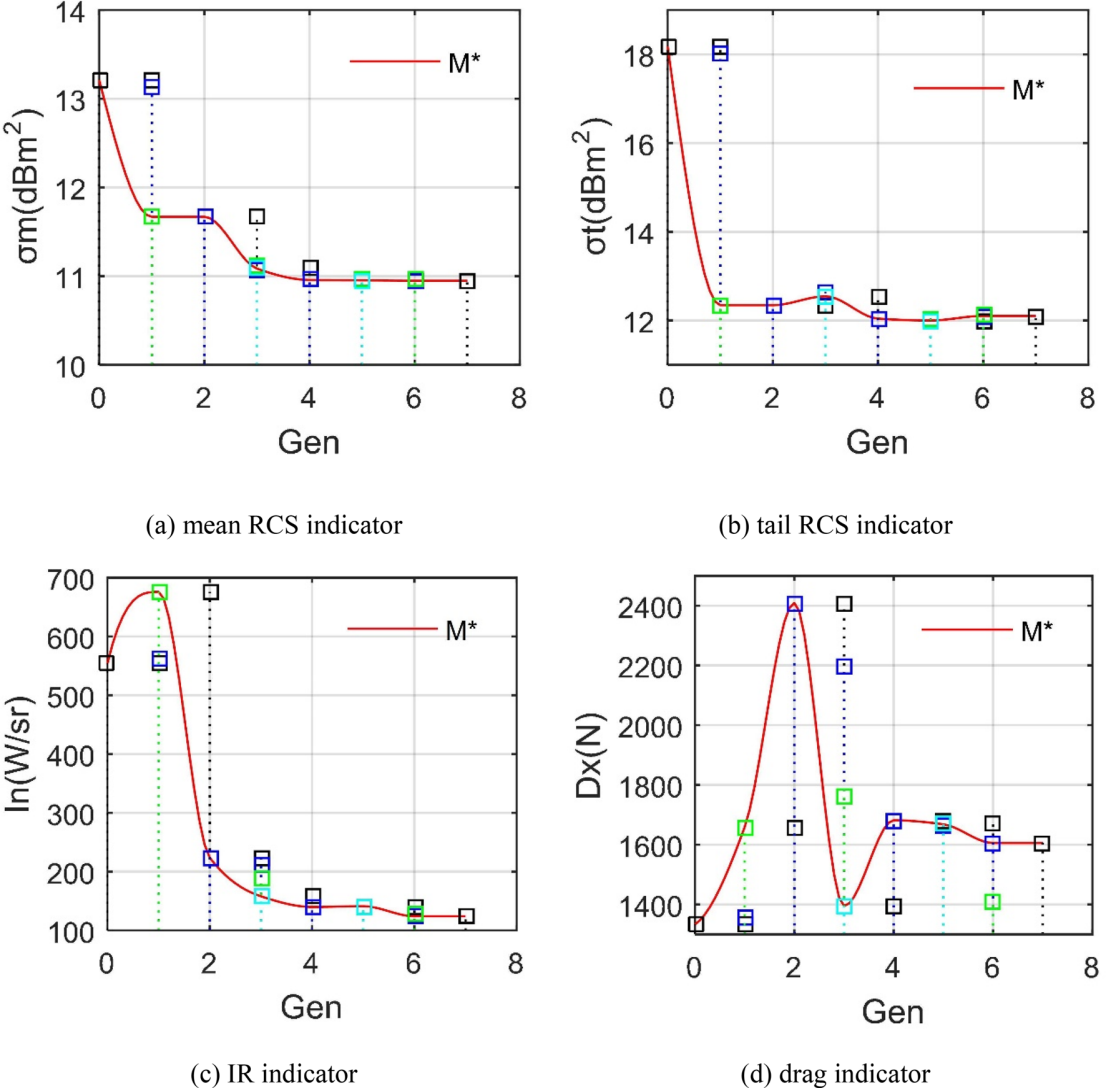
History chart of various performance indicators.

Figure 16 supports that the three stealth indicators have been improved overall, and the aerodynamic indicator has made a little sacrifice. For the mean RCS of M*, *σ*_m_ generally maintains a steady downward trend because both ranking factors and priorities are beneficial to this radar stealth indicator. The tail RCS indicator has been greatly reduced, which is conducive to the rearward stealth of the exhaust system. As shown in Table 4, *σ*_t_ is reduced from greater than 18 dBm^2^ to 12.1045 dBm^2^, the reduction is 6.0763 dBm^2^. This is mainly because of the reasonable choice of exhaust port model and the optimization of nozzle height. The infrared signature indicator of M* shows a trend of increasing first and then decreasing sharply. Many factors contributed to this process, including the exhaust port model, nozzle series and lower baffle, where *I*_n_ indicator has been reduced by 430.2313 W/sr. The drag performance of the individual fluctuated greatly throughout the process with a maximum difference of 1076.172 N, while the *D*_*x*_ indicator of M*increased by 272.9023 N from 1332.9243 N. Overall, a little drag sacrifice here is also acceptable. These results indicate that CDM is satisfactory for improving the radar/infrared stealth characteristics of the aircraft's exhaust system.

**Table 4:** Aircraft main performance comparison before and after CDM.

	*σ*_m_ (dBm^2^)	*σ*_t_ (dBm^2^)	*I*_n_ (W/sr)	*D*_*x*_(N)
*m*_0_	13.2120	18.1808	554.3546	1332.9243
M*	10.9492	12.1045	124.1233	1605.8266

The changes in the design parameters or sub-models of the exhaust system are shown in Table 5, where all the design factors here have been improved under the comprehensive evaluation of CDM. The exhaust port model changed from a simple round tube to a rectangular design with upper and lower triangular baffles. The nozzle of the exhaust system was upgraded from single-stage injection to two-stage injection. The size of the upper and lower baffles, the height and width of the nozzle have also changed. These results indicate that CDM is clearly effective for the overall design of this aircraft exhaust system.

**Table 5:** Comparison of main design parameters or sub-models of aircraft before and after CDM.

	*M*_ex_	*N*_s_	*B*_d_(mm)	*H*_n_(mm)	*W*_n_(mm)	*B*_u_(mm)
*m*_0_	*M*_ex1_	1	*X*_v_ = 500	330	800	*X*_u_ = 300
M*	*M*_ex3_	2	*X*_v_ = 1150	300	950	*X*_u_ = 400

**Figure 17:**
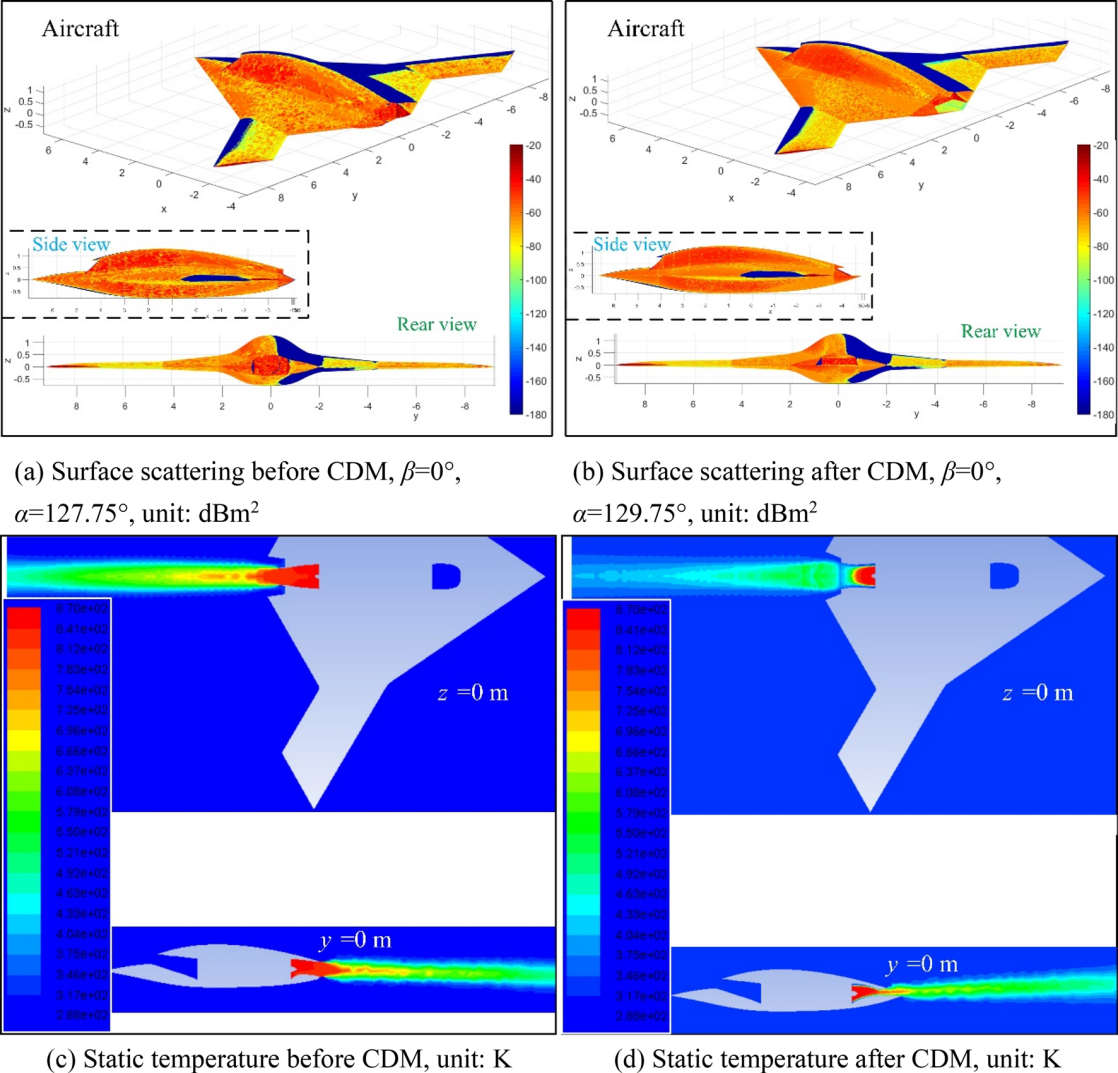
Performance comparison of the aircraft before and after CDM, drawn using Microsoft Visio 2007 and MATLAB 2015A.

Figure 17 presents that the optimized aircraft model has made significant improvements in surface electromagnetic scattering characteristics and plume temperature field. For the Fig. 17a, the cut surface at the tip of the wing, the wall near the tail nozzle and the upper middle part of the fuselage show the most obvious red under the current incident wave, because at this time, the angle between the radar wave and the normal direction of the wing tip is very small, so that the surface here does not have a good ability to deflect the radar wave. The tail nozzle adopts a conventional round tube design, and the nearby end face and baffle form a strong scattering source, that is, a right-angle dihedral angle, which leads to a high RCS level here. For the Fig. 17b, at this time, the incident azimuth angle of the radar wave has increased by 2°, and the distribution of strong scattering sources on the surface of the aircraft is still similar compared with the initial model, while the performance of RCS near the tail nozzle has been obviously changed, because the lower baffle of the nozzle of the optimized model adopts a shielding design that blends with the curve of the fuselage tail, the baffle on the side of the nozzle does not form a right-angle dihedral angle with the nozzle end surface, the upper baffle is extended from the surface of the fuselage and cut off with a triangular outer contour, the combined effect of these measures makes the optimized aircraft model have a good low electromagnetic scattering level. For the Fig. 17c, it can be found that there is a large-area high-temperature core zone with a temperature range of 696 K ~ 841 K in the tail flame outside the nozzle of the initial model, where the static temperature of the space in the exhaust nozzle pipe is basically above 812 K, because the exhaust system at this time uses a single-stage ejection method, which makes the high-temperature wake jet from the center cone unable to be directly cooled until it reaches the edge of the nozzle. For the Fig. 17d, it can be noticed that the core area of the plume whose static temperature exceeds 801 K is confined in the space of the jet pipe, where the static temperature of the tail flame outside the baffle under the nozzle is basically below 648 K, because the optimized exhaust system has a design based on the principle of secondary ejection, coupled with the combined effect of nozzle height, outer width, and upper and lower baffles, the temperature field of the entire aircraft's tail flame is very popular. These results show that the CDM based on the Pareto solution of the ranking factor is feasible and effective to improve the electromagnetic scattering and infrared radiation of this aircraft.

## Conclusions

By studying the radar cross-section and infrared radiation characteristics of this aircraft exhaust system, the relevant parameters and sub-models are fully designed, and the following conclusions can be obtained:

1. The exhaust port model, lower baffle and nozzle height are the main factors affecting the two RCS indicators of this aircraft model. The design of the upper baffle has a greater impact on the drag indicator than on the other three indicators.
2. The order that has the greatest influence on the infrared radiation indicator is the nozzle stages, the exhaust port model, the lower baffle, the nozzle outer width and the nozzle height. For the drag indicator, the order is the lower baffle, nozzle stages, exhaust port model, nozzle height and nozzle outer width.
3. CDM can effectively improve the radar/infrared stealth performance of the aircraft exhaust system while sacrificing some aerodynamic drag. This mainly benefits from the Pareto solution based on ranking factors and priority comparison.

The main contribution of this paper is the presentation of CDM and ranking factors. The former is to improve the target’s aerodynamic/stealth comprehensive performance, and the latter is to establish relatively good individuals in the Pareto solution.
